# Detection of Critical Parts of River Crab Based on Lightweight YOLOv7-SPSD

**DOI:** 10.3390/s24237593

**Published:** 2024-11-28

**Authors:** Guoai Fang, Yu Zhao

**Affiliations:** College of Engineering Science and Technology, Shanghai Ocean University, Shanghai 201306, China; m220851437@st.shou.edu.cn

**Keywords:** crab processing, target detection, YOLOv7-tiny, lightweight, DepGraph pruning

## Abstract

The removal of back armor marks the first stage in the comprehensive processing of river crabs. However, the current low level of mechanization undermines the effectiveness of this process. By integrating robotic systems with image recognition technology, the efficient removal of dorsal armor from river crabs is anticipated. This approach relies on the rapid identification and precise positioning of the processing location at the crab’s tail, both of which are essential for optimal results. In this paper, we propose a lightweight deep learning model called YOLOv7-SPSD for detecting river crab tails. The goal is to accurately determine the processing location for the robotic removal of river crab back armor. We start by constructing a crab tail dataset and completing the data labeling process. To enhance the lightweight nature of the YOLOv7-tiny model, we incorporate the Slimneck module, PConv, and the SimAM attention mechanism. These additions help achieve an initial reduction in model size while preserving detection accuracy. Furthermore, we optimize the model by removing redundant parameters using the DepGraph pruning algorithm, which facilitates its application on edge devices. Experimental results show that the lightweight YOLOv7-SPSD model achieves a mean Average Precision (mAP) of 99.6% at a threshold of 0.5, an F1-score of 99.6%, and processes frames at a rate of 7.1 frames per second (FPS) on a CPU. Compared to YOLOv7-tiny, the improved model increases FPS by 2.7, reduces GFLOPS by 74.6%, decreases the number of parameters by 71.6%, and lowers its size by 8.1 MB. This study enhances the deployment of models in river crab processing equipment and introduces innovative concepts and methodologies for advancing intelligent river crab deep processing technology.

## 1. Introduction

The Chinese mitten crab (*Eriocheir sinensis*), also known as the river crab, possesses significant 4nutritional and economic value [1,2]. In China, river crabs are mainly used for two purposes: fresh food and deep processing. As of 2022, the total freshwater aquaculture production of river crabs reached 815,000 tons. Of this total, the fresh food market accounted for 98.89%, while the deep processing market made up just 1.11% [3]. In comparison to the fresh food market, the deep-processing market for river crabs is significantly underdeveloped, largely due to the complex structure of the crabs. The processing methods used are mostly partial and cumbersome. These methods involve multiple steps, including removing the dorsal shell, crab legs, and crab yolk, as well as separating the shells from the meat of various parts of the crab. Moreover, the majority of these processes rely on manual shelling instruments (e.g., hammers, shovels, needles, forks, and scissors) for separating different crab parts [4,5], which results in high labor intensity, low processing efficiency, and a risk of contamination of the crab meat. The challenges of high labor intensity, low processing efficiency, and the risk of crab meat contamination continue to be significant issues. To improve the processing efficiency of river crabs, most current research focuses on enhancing devices designed for specific parts of the crab. This includes tools for extruding shells and separating meat from crab feet, cutting devices for the crab body, and mechanisms for adsorbing and removing the dorsal armor [6,7]. Although these devices enhance specific processing methods for river crabs, they only focus on certain aspects of the process, which makes it difficult to achieve precise processing positions. As a result, the outcomes of the processing are less than ideal, and the level of automation is low. This, in turn, significantly hampers the development of the deep-processing industry for river crabs [8]. Recent advancements in robotics and image recognition technology have made it possible to simulate the manual processing of river crabs. A significant challenge in this process is the quick and accurate identification of key points for each step, such as the joints of the crab legs and pincers that connect to the crab body, the tail of the river crab, and the yolk pipeline linked to the dorsal carapace. Among these key points, detecting the crab’s tail is the first step in the deep processing of river crabs, as it is necessary for the removal of the dorsal armor. Therefore, it is essential to clarify the key position for the removal of the dorsal armor in river crabs and to explore automated and intelligent methods for processing them into parts. This approach will improve processing efficiency and support the sustainable development of river crab processing equipment.

In recent years, the rapid development of deep learning in image recognition has led to the widespread application of machine vision in the deep processing of aquatic products, thereby enhancing the accuracy of processing and promoting the automation of aquatic processing [9]. In 2018, Wang et al. pioneered the integration of image analysis into automated crab processing by proposing a convolutional neural network-based computer vision algorithm, achieving a remarkable accuracy of 97.67% in detecting dorsal fin nodes of green crabs [10]. Subsequently, Wang et al. introduced a method to classify the quality of river crabs by combining image processing techniques, genetic algorithms, and neural networks from BP, achieving an accuracy rate of 92.7% [11]. While these methods represented significant strides in the intelligent processing of aquatic products, challenges such as large network models, slower detection speeds, and insufficient real-time performance persisted. In recent years, the YOLO (You Only Look Once) algorithm [12] and advancements in model lightweighting technology have substantially improved the efficiency of intelligent processing. Ye et al. enhanced the YOLOv5 model by integrating the CA attention mechanism and Bottleneck Transformer, addressing the labor intensity and inefficiency of traditional crayfish sorting and providing a rapid automated solution [13]. Chen replaced the CSP-Darknet53 backbone of YOLOv4 with GhostNet, added the SE attention mechanism and CIoU, and achieved automatic sorting of both shelled and peeled shrimp with a lightweight model achieving a mAP of 98.5%, thereby enhancing the automation of shrimp sorting [14]. Chen et al. adopted the PP-LCNet architecture in YOLOv5 to address the high labor intensity and inefficiency in traditional manual crayfish sorting, enabling efficient and automated sorting. Furthermore, by employing PP-LCNet as the backbone of YOLOv5, they classified the quality of South American white shrimp by incorporating a DepthSepConv module and replacing the SiLU activation function, achieving a mAP of 98.5% with 4.8 M parameters and 9.0 GFLOPs [15]. Although these methods have successfully reduced computational load, the resulting models may still be too heavy for some applications. Additionally, the process of making these models lightweight often leads to compromises in their detection capabilities. Therefore, there is an urgent need to explore new strategies and techniques to enhance the detection capabilities of lightweight YOLO networks. This will help improve both accuracy and real-time performance in aquatic processing, addressing the demands of current lightweight edge devices.

This paper explores ways to improve the intelligence of equipment used for processing river crabs and to achieve precise part processing. The main focus is on the crab tail, which is the primary area for removing the dorsal armor. The paper examines efficient methods for identifying and localizing this area using machine vision. To address this, we propose a lightweight deep learning model called YOLOv7-SPSD, which is based on YOLOv7-tiny. It achieves initial lightweighting by introducing the Slimneck module [16], PConv [17], and SimAM attention mechanism [18]. Additionally, the model utilizes the DepGraph pruning algorithm [19] to remove redundant parameters, further enhancing model lightweighting to meet the requirements of edge devices. This approach opens up possibilities for the application of lightweight networks in river crab processing equipment.

## 2. Materials and Methods

### 2.1. Data Acquisition and Preprocessing

**Figure 1:**
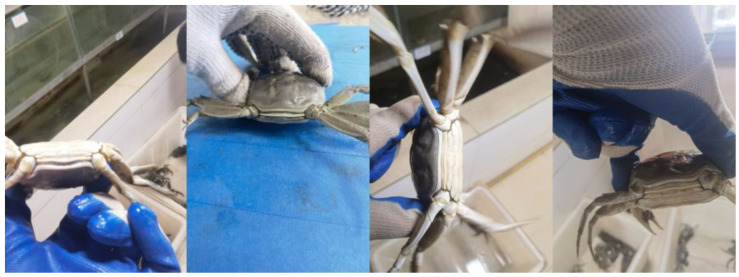
Some of the original images.

The crab tail data for this study were collected in October 2023 in Rulin Town, Jintan, Changzhou, Jiangsu Province. To enhance the representativeness of the samples, the applicability of the model, and the compatibility of the equipment, a range of devices was used to capture images. These devices included the Huawei Mate40 Pro, iqoo11 smartphones, and an iPad. To ensure the representativeness of the sample data, crabs weighing between 100 and 200 g were randomly selected from uniform breeding pools of both males and females. Due to challenges in capturing larger live crabs, both handheld and fixed desktop shooting methods were employed. Pictures were taken at distances ranging from 0.1 m to 1 m, using random shooting angles. Unclear or damaged images were discarded, resulting in a total of 750 JPG format pictures, as shown in Figure 1.

To reduce data variability arising from multiple device acquisitions, all image lengths were fixed at 640 pixels to enhance the model’s robustness. Additionally, factors such as lighting, obstacles, and human influences affect actual river crab processing. To enhance the model’s robustness and mitigate overfitting resulting from insufficient training data, the dataset is expanded through data augmentation, enriching the dataset and improving the generalization ability of the training model. Initially, the original images were labeled using LabelImg. Various augmentation methods, including random rotation, random translation, vertical flip, random hue saturation adjustment, addition of pretzel noise, and Gaussian noise, were employed. At least two augmentation methods were applied to each image. This process resulted in a total of 3000 images, which constituted the crab tail dataset. These images were then randomly divided into training, validation, and test sets in a ratio of 7:1:2.

### 2.2. YOLOv7-Tiny Base Model

**Figure 2:**
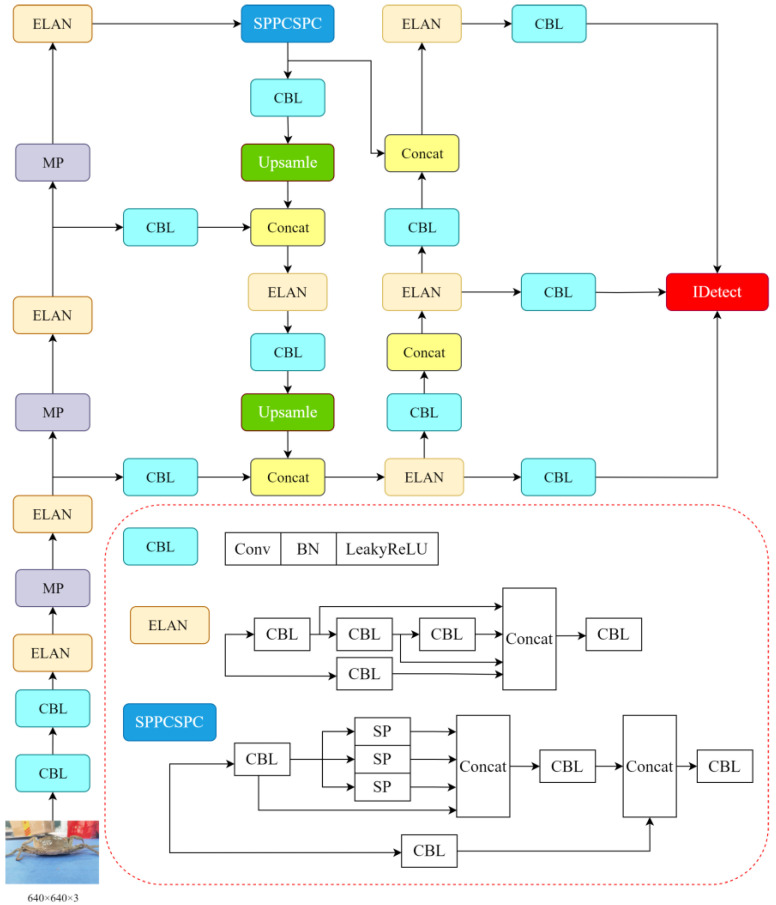
YOLOv7-tiny network structure.

YOLOv7-tiny is a lightweight variant of the YOLOv7 model [20], designed specifically for edge devices, and comprises the main trunk, neck, and head, as shown in Figure 2. It retains the scaling strategy of the YOLOv7 composite model and enhances the Efficient Remote Aggregation Network (ELAN), while reducing the number of convolutions in the ELAN [21], MP, and SPPCSPC modules. Although the ELAN module has been optimized for efficiency in YOLOv7-tiny compared to the standard YOLOv7, the increased number of ELAN modules somewhat heightens the network’s computational load.

### 2.3. Improved Lightweight YOLOv7-SPSD

#### 2.3.1. Lightweight NECK: Slimneck

Typically, lightweight network designs favor the use of depthwise separable convolution (DSC), which offers high computational efficiency. However, during the computation, DSC separates the channel information from the input image, which significantly reduces the capabilities for feature extraction and fusion. To address this issue, a hybrid convolution method called GSConv is introduced, based on relevant research in lightweight network design. Compared to conventional convolution, GSConv maximizes the retention of hidden connections between channels while maintaining low time complexity. This approach minimizes information loss and accelerates computation [22]. This approach effectively combines the benefits of standard convolution (SC) and DSC.

**Figure 3:**
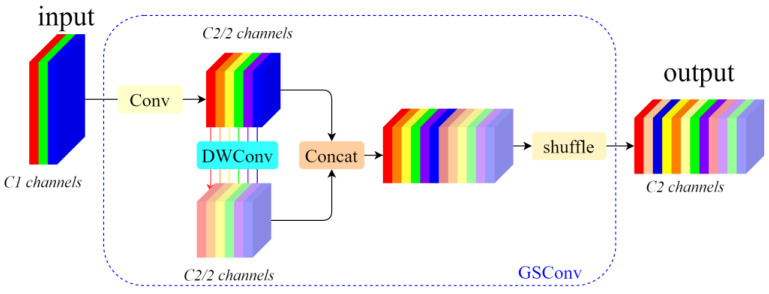
GSConv structure.

The GSConv module primarily consists of four components: the Conv module, the DWConv module, the Concat module, and the Shuffle module. As shown in Figure 3, we assume that the input feature map has C1 channels. In this setup, depth-separable convolution utilizes half of these channels, while the other half is processed using regular convolution. The outputs from the two types of convolution are then concatenated to facilitate feature fusion. After this, the information produced by SC is blended with various parts of the information generated by DSC. The result is an output feature map with a total of C2 channels. The expression for the GSConv module is as follows:

(1) $$X_{out}=f_{shuffle}\left(cat\left(f_{conv}\left(X_{in}\right),f_{dsc}\left(f_{conv}\left(X_{i}\right)\right)\right)\right)$$

**Figure 4:**
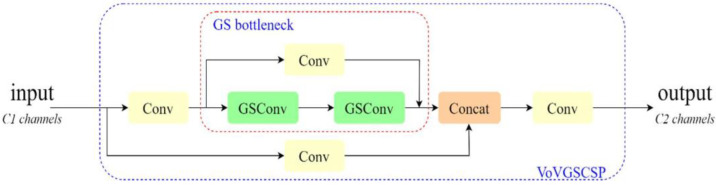
VoVGSCSP structure.

Although GSConv effectively reduces redundant information in the feature maps of the crab tail detection model, it does not significantly decrease inference time while maintaining accuracy. To address this limitation, the VoVGSCSP module replaces the ELAN module in the neck network section. The VoVGSCSP is created by combining the modules shown in Figure 3 using a one-time aggregation method, and its structure is illustrated in Figure 4. This approach decreases both computational and architectural complexity, further minimizing the model’s memory footprint and facilitating deployment on edge devices with limited computational resources.

#### 2.3.2. Lightweight Convolution: Pconv

A large number of ELAN modules are used in the YOLOv7-tiny backbone network to address the elongation of the shortest gradient paths caused by an excessive number of transition layers. While the ELAN modules help mitigate the reduction in overall network accuracy and potential convergence issues when scaling the model, their effectiveness in terms of the number of network parameters and computational demand remains inadequate. To tackle this problem, this paper introduces PConv (Partial Convolution) into the ELAN model, resulting in the creation of the ELAN-P module. This adaptation not only reduces computational redundancy but also preserves the performance of the ELAN structure and decreases memory access.

**Figure 5:**
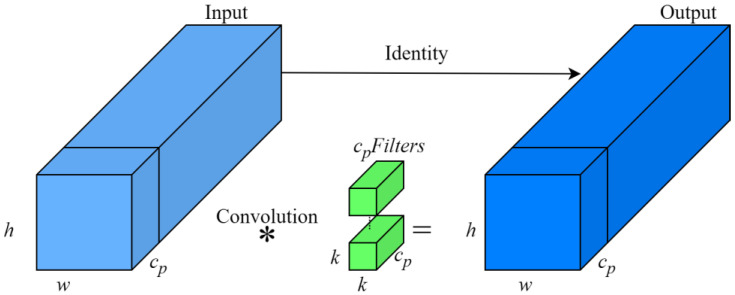
PConv structure.

Unlike conventional convolution operations, PConv performs selective feature extraction by focusing on specific input channels while leaving the others unchanged. The structure of PConv is illustrated in Figure 5. In the YOLOv7-tiny backbone network, which includes the ELAN-P module, PConv is used for extracting features related to crab tails. It computes selected channels as representatives of the entire feature map, thereby reducing computational load while maintaining generalizability. The computation of FLOPs and storage accesses for the PConv module can be expressed as follows:

(2) $$h\times w\times k^{2}\times c_{p}^{2}$$

(3) $$h\times w\times 2c_{p}+k^{2}\times c_{p}^{1}\approx h\times w\times 2c_{p}$$

In this equation, h represents the height of the feature map, w represents the width of the feature map, k represents the convolutional kernel size, and $c_{p}$ represents the number of output channels. When the partial ratio $r=c_{p}/c=1/4$, the FLOPs for PConv amount to merely 1/16th of those for a standard Conv, and its memory accesses are only 1/4th compared to a standard Conv.

In PConv convolution, only the $c_{p}$ channels are utilized for spatial feature extraction, while the remaining channels, which are unchanged, facilitate the transfer of feature information between channels. Compared to the ELAN network, the ELAN-P network significantly reduces computation and memory access requirements. The overall structure of the network has been effectively optimized, leading to an increase in the speed of inference.

#### 2.3.3. Parameter-Free Attention Mechanism: SimAM

**Figure 6:**
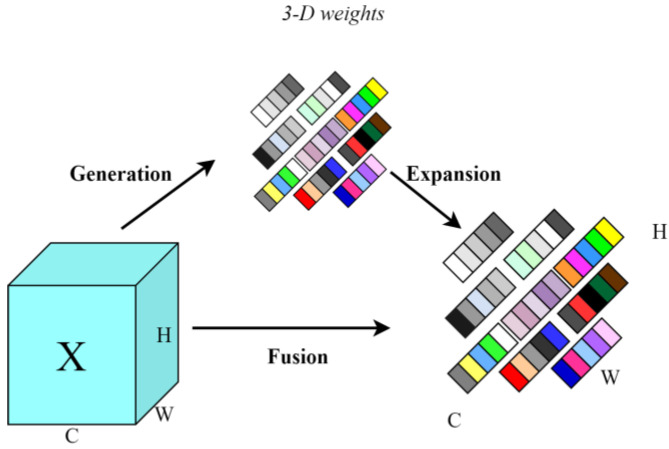
SimAM attention mechanism structure.

Attention mechanisms are widely utilized in deep learning to enhance feature extraction and target useful objects. However, most current attention modules typically assign weights along the channel dimensions, thereby improving the performance of the model and introducing additional parameters [23]. SimAM is a parameter-free attention mechanism that dynamically assigns 3D attention weights to the feature graph, enhancing the model’s capability to extract features. This mechanism calculates attention weights directly from the feature map without introducing any additional parameters, which increases efficiency and reduces model complexity. The structure of SimAM is illustrated in Figure 6.

The SimAM attention mechanism identifies critical neurons by evaluating the linear separability between them and assigning higher priorities to those neurons. Informational neurons display distinct firing patterns compared to their neighboring neurons and exhibit spatial inhibition effects. A key method for localizing these neurons involves measuring the linear separability of a target neuron from its counterparts. The energy function of each neuron reaches its minimum value when the target neuron is linearly separable from all other neurons within the same channel. The energy function for each neuron is calculated using the following equation:

(4) $$e_{t}\left(w_{t},b_{t},y,x_{i}\right)={\left(y_{t}-\hat{t}\right)}^{2}+\frac{1}{M-1}\overset{M-1}{\underset{i=1}{\sum }}{\left(y_{0}-{\hat{x}}_{i}\right)}^{2}$$

Among them: $\hat{t}=w_{t}t+b_{t}$; $\hat{x}=w_{t}x_{i}+b_{t}$.

M represents the total number of neurons, t represents the target neuron, $x_{i}$ represents other neurons, $w_{t}$ represents the weights, and $b_{t}$ represents the bias. To simplify the computations, scalars $y_{t}$ and $y_{0}$ are assigned binary labels, and the regular term λ is incorporated, subsequently recombining these elements to define the final energy function:

(5) $$\frac{1}{M-1}\overset{M-1}{\underset{i=1}{\sum }}{\left(-1-\left(w_{i}x_{i}+b_{t}\right)\right)}^{2}+{\left(-1-\left(w_{t}t+b_{t}\right)\right)}^{2}+\lambda w_{t}^{2}$$

Solving the above equations yields the weights and the bias as follows:

(6) $$w_{t}=-\frac{2\left(t-\mu_{t}\right)}{{\left(t-\mu_{t}\right)}^{2}+2\sigma t^{2}+2\lambda }\;$$

(7) $$b_{t}=-\frac{1}{2}\left(t+\mu_{t}\right)w_{t}$$

$\mu_{t}$ and σ denote the mean and variance, respectively, of each neuron in that channel except for neuron t, which ultimately leads to the formula for the minimum energy:

(8) $$e_{t}^{*}=\frac{4\left(\hat{\sigma }+\lambda \right)}{{\left(t-\hat{\mu }\right)}^{2}+2{\hat{\sigma }}^{2}+2\lambda }$$

From Equation (8), it is evident that the smaller the value of the energy function, the greater the linear separability between the neuron t and other neurons. The entire attention module operates under the guidance of this energy function, minimizing excessive computational and tuning efforts. Neural networks can be enhanced by manipulating individual neurons and incorporating this concept of linear differentiability into the network architecture. Incorporating SimAM into the YOLOv7-tiny model enhances crab tail detection performance without adding to the computational complexity of the model.

#### 2.3.4. DepGraph Pruning

Structural pruning is a neural network optimization technique that involves removing entire channels [24], filters, or layers from a network to significantly reduce its computational and memory demands. Unlike weight pruning [25], which nullifies individual weights based on their magnitude, structural pruning targets entire structural components of the network. It achieves greater sparsity than weight pruning by eliminating redundancies, such as entire channels or filters, that contribute minimally to network performance. However, structural pruning necessitates careful design and analysis to ensure that the pruned network maintains the essential representational capacity to perform necessary tasks with high accuracy. To preserve the accuracy of the original unpruned network as much as possible, this paper utilizes DepGraph generic structural pruning.

**Figure 7:**
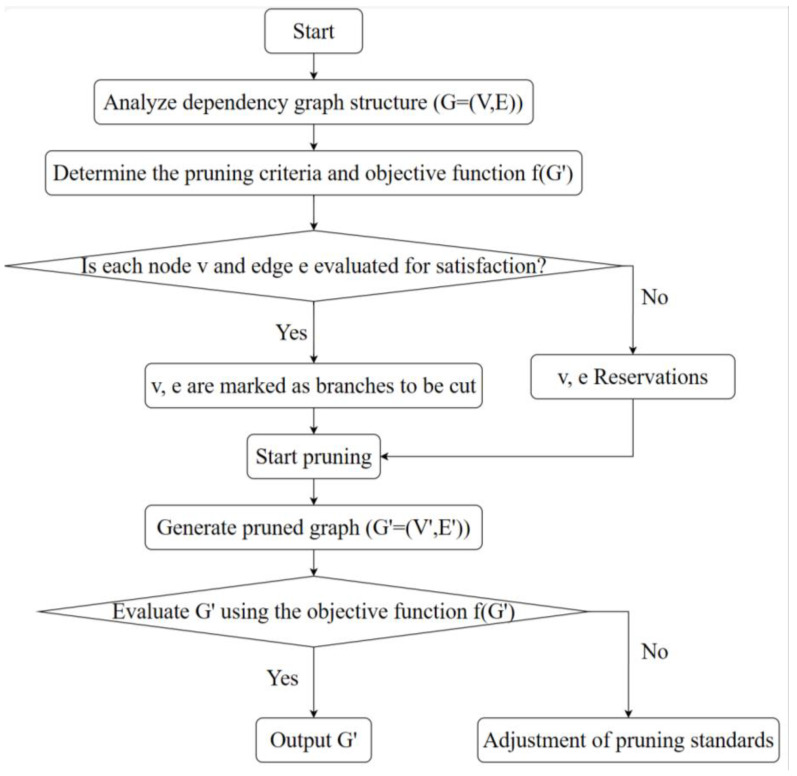
DepGraph pruning workflow.

DepGrap pruning leverages the local dependencies between neighboring layers in neural networks to create a dependency graph, through which the network model is constructed for structural pruning. Thus, DepGrap pruning is essentially an optimization problem constructed through a dependency graph, where the dependency graph is depicted as a directed graph $\;G=\left(V,E\right)$, with V representing the vertices for entities (such as tasks, concepts, etc.) and *E* representing the edges for dependencies or connections. The objective of pruning is to identify a subgraph $G^{\prime }=\left(V^{\prime },E^{\prime }\right)$ where $V^{\prime }\subseteq V$ and $E^{\prime }\subseteq E$, ensuring that $G^{\prime }$ retains essential structural information for a specific objective while eliminating redundant complexity, as illustrated in the detailed workflow in Figure 7.

#### 2.3.5. The YOLOv7-SPSD Model

**Figure 8:**
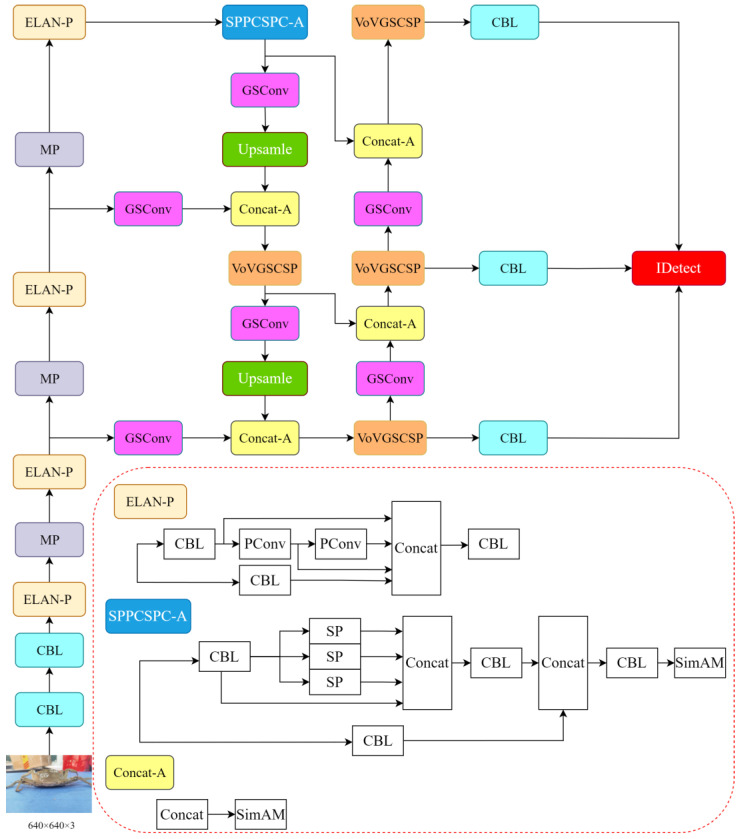
Improved network structure with lightweighting of modules.

This paper presents a comprehensive analysis and experimentation that leads to the proposal of integrating GSConv and VoVGSCSP to replace the computationally intensive ELAN module in the neck section of the model. In the trunk section, the incorporation of the lighter-weight PConv allows us to preserve the beneficial feature extraction capabilities of the ELAN model while reducing computational redundancy, without compromising performance. To address specific detection limitations of Slimneck, we employ the SimAM attention mechanism in the SPPCSPC and Concat layers. This ensures the model’s overall effectiveness without introducing unnecessary parameters. Compared to the original model, this enhanced version achieves significant lightweighting while maintaining detection performance. The refined network structure is illustrated in Figure 8. Furthermore, the improved model undergoes parameter pruning using DepGraph to eliminate redundancy, resulting in the development of the YOLOv7-SPSD model.

## 3. Results and Discussion

### 3.1. Experimental Environment

In this paper’s experiments, experiments were conducted using the PyTorch2.0 deep learning framework on a 13th Gen Intel(R) Core(TM) i5-13500HX@2.50 GHz (16 GB RAM, Intel Corporation, Santa Clara, United States) and NVIDIA GeForce RTX4060 Laptop GPU (8 GB RAM, NVIDIA Corporation, Santa Clara, United States) operating under a Windows 11 operating system hardware platform. The entire training process was configured for 300 cycles, with a batch size of 16; the input image size was 640 × 640; the initial learning rate was set at 0.1; a stochastic gradient descent optimizer was utilized and no pretrained weights were loaded.

### 3.2. Indicators for Evaluation

In this paper, P (precision), R (recall), F1 (F1-score), AP (average precision), mAP (mean average precision), the model parameters, FLOPs (floating point operations), GFLOPS and model size were utilized as evaluation metrics. As an evaluation metric, the IOU (intersection over union) threshold was set at 0.65. Precision is defined as the ratio of correctly identified river crab parts to the total number of detected river crab parts. Recall is the ratio of correctly detected river crab parts to the total number of parts in the dataset. F1 is the harmonic mean of precision and recall. Generally, a higher F1 indicates greater model stability. mAP evaluates overall performance across various confidence thresholds. Additionally, the number of parameters, computational requirements and model size assess complexity of the model.

(9) $$P=\frac{TP}{TP+FP}$$

(10) $$R=\frac{TP}{TP+FN}$$

(11) $$F_{1}=\frac{2\times P\times R}{P+R}$$

(12) $$A_{P}=\int_{0}^{1}P\left(R\right)dR\times 100\%$$

(13) $$mAP=\frac{1}{M}\sum_{k=1}^{M}A_{P}\left(k\right)\times 100\%$$

where TP represents a true positive, FP a false positive, TN a true negative, and FN a false negative.

### 3.3. Ablation Experiment

To verify the effectiveness of Slimneck, PConv, SimAM, and DepGraph pruning, ablation experiments were carried out using the YOLOv7-tiny model and the results are shown in Table 1.

As shown in Table 1, replacing the neck component of the original YOLOv7-tiny model with Slimneck resulted in a decrease of 0.8 GFLOPS, 0.3 MB in model size, and a 3.5% reduction in parameters. While the mAP0.5 remained unchanged, there were slight decreases in P, R, and F1. Subsequently, integrating the complex ELAN module in the backbone with PConv retained the advantages of the ELAN module while significantly reducing the computational overhead for feature extraction. After modifying the Slimneck structure and replacing the ELAN module with the ELAN-P module, although mAP0.5 decreased by 0.1% compared to the original model, GFLOPS was reduced by 3.9, model size was reduced by 2.8 MB, and parameters decreased by 25.1%. These changes compensated to a certain extent for the decreases in P, R, and F1, further enhancing the feature extraction ability for the crab tail. Given the limited feature fusion capability of Slimneck, the inclusion of the SimAM attention mechanism in the SPPCSPC and each Concat module within the neck segment served to enhance Slimneck’s feature fusion capacity. Following the integration of SimAM, there was no increase in GFLOPS, size, or parameters. Both mAP0.5 and F1 achieved the same levels of accuracy as the baseline model, with recall increasing by 0.2%. Therefore, by concurrently employing Slimneck, ELAN-P, and SimAM, the model maintains both accuracy and precision while achieving weight reduction.

**Figure 9:**
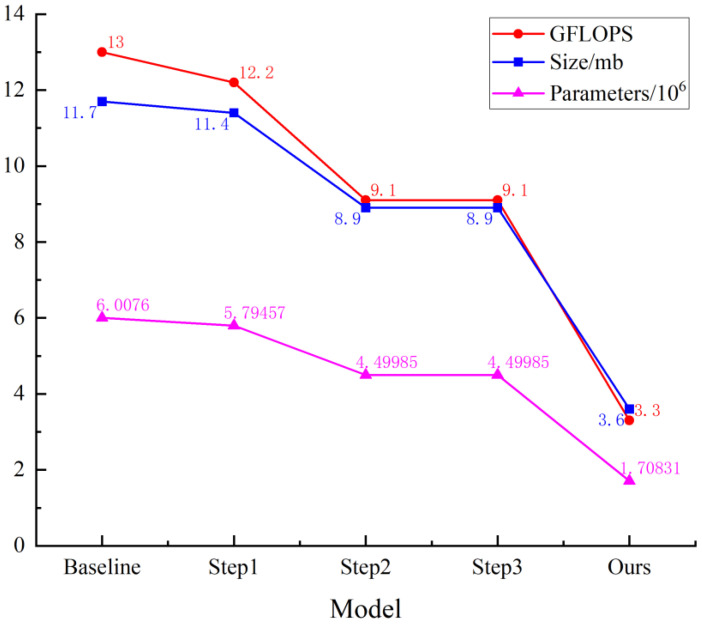
Variation of GFLOPS, Size, and Parameters in ablation experiment.

Finally, the model, which integrates the three modules, was pruned using the DepGraph method to eliminate redundant parameters and further reduce its weight. After pruning, mAP0.5 increased by 0.1%, recall improved by 0.2%, and the F1 increased by 0.1%; GFLOPS decreased by 74.6%, size by 8.1 MB, and parameters by 71.6%. The ablation experiment results are illustrated in Figure 9, showing notable reductions in GFLOPS, size, and parameters compared to the baseline model. The improvements brought by the three modules, along with the DepGraph pruning algorithm, play a significant role in preserving the detection performance of the YOLOv7-SPSD model, all while decreasing its parameter count, computational load, and overall size.

### 3.4. Pruning Experiment

In this study, we applied the DepGraph algorithm to prune the enhanced model. We established a pruning multiplier parameter and iteratively adjusted it to find the optimal value. This process allowed us to compress the model to its maximum potential while maintaining consistent experimental conditions. The results of the pruning and compression experiments are presented in Table 2.

**Figure 10:**
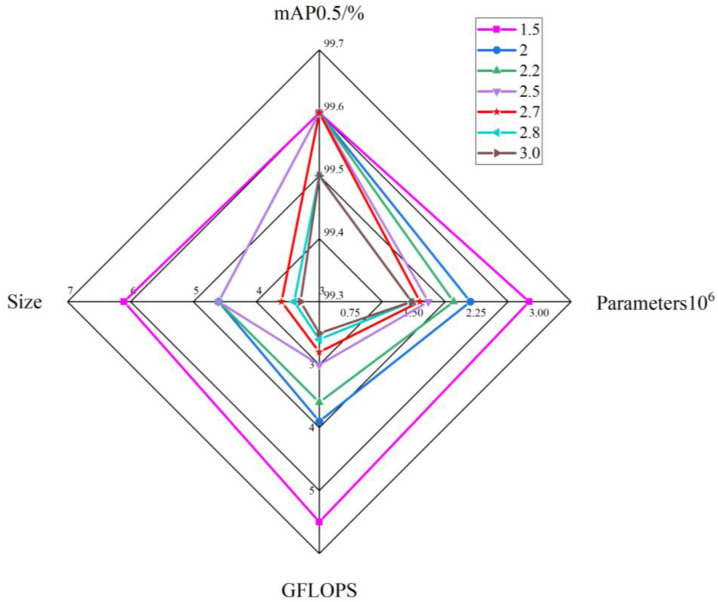
Radar plot comparing the performance of models with different compression multiples.

Analysis of the data reveals that mAP0.5 remains consistent at 99.6% for compression multipliers of 1.5, 2, 2.2, 2.5, and 2.7. However, at a compression multiplier of 2.8, mAP0.5 slightly decreases to 99.5%, representing a decline of 0.1%. To enhance the accuracy of crab tail detection, we determined through experimentation that the optimal upper limit for the pruning multiplier is 2.7. Furthermore, Figure 10 illustrates radar plot analyses of the data from our table. In these plots, the largest graph area corresponds to a multiplier of 2.7, indicating that this is the most effective pruning multiplier for the DepGraph algorithm.

To evaluate the performance of the DepGraph algorithm, this study compares the maximally compressed model produced by DepGraph with several other popular pruning algorithms, including L1, Lamp, Slim, and Taylor. All experiments utilized enhanced lightweight base models, which were pruned at various proportions under the same conditions. The results of these experiments are presented in Table 3.

**Figure 11:**
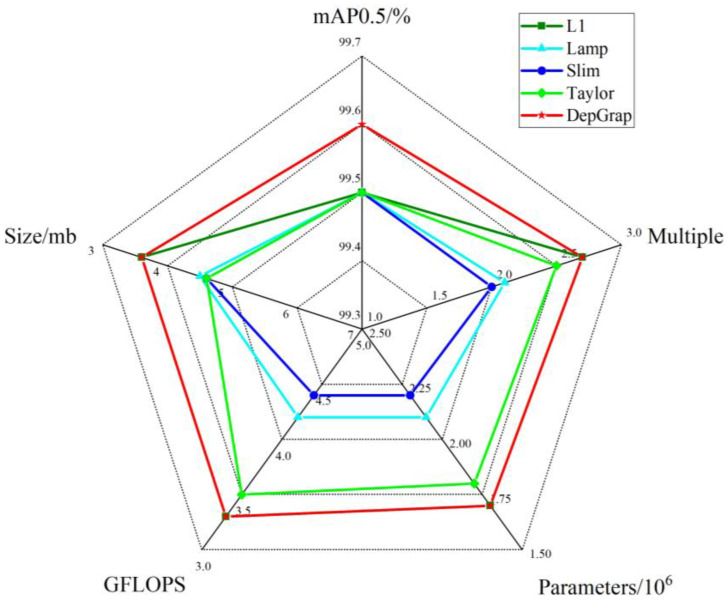
Radar plot comparing the comprehensive performance of different pruning algorithms.

Analysis of the table reveals that the DepGraph pruning algorithm offers significant advantages in terms of detection accuracy, parameter count, computational demand, model size, and compression ratios. The DepGraph algorithm maintains 99.6% mAP0.5 even when compressed by a factor of 2.7, marking a 0.1% increase in performance before pruning. Furthermore, the post-pruning parameter count, computational requirements, and model size are more favorable compared to those of other pruning algorithms. While some other methods achieve a degree of reduction, they typically experience a significant decline in accuracy as the compression ratio increases. The radar plot analysis shown in Figure 11 demonstrates that the area representing the model pruned with the DepGraph algorithm is the largest, indicating superior performance across all metrics in comparison to other algorithms.

### 3.5. Model Comparison Experiment

To evaluate the effectiveness of the YOLOv7-SPSD model in detecting crab tails, this study conducted a comprehensive comparative analysis of major target detection networks, including the YOLOR, YOLOv5, YOLOv7, and YOLOv8 series. Throughout the training process, all networks used the same dataset and did not utilize any official default weights. Additionally, to assess the performance of the various models on edge devices, the CPU was employed to measure the FPS of the networks. The image input size for all models was set to 640 × 640, and a total of 600 images from the set of experiments were fully evaluated. The results of each target detection model upon completion of the tests are presented in Table 4.

From the analysis presented in the table, it is evident that YOLOR-CSP and YOLOv7 achieve higher mAP0.5 and F1-score metrics. However, these models are characterized by substantial model parameters and high computational intensity, resulting in low FPS on CPUs. This limitation complicates their deployment on edge computing devices with limited GPU resources. On the other hand, YOLOv5s and YOLOv8n demonstrate effectiveness in detecting crab tails. Nevertheless, their overall performance is inferior to that of YOLOv7-tiny. Notably, YOLOv5-Lite, with its fewer parameters, reduced computational demands, and smaller model size, exhibits significantly lower precision, recall, mAP0.5, and F1-score compared to other networks. While YOLOv5n achieves a higher FPS, its mAP0.5 and F1-score are 0.3% and 1.6% lower, respectively, than those of YOLOv7-SPSD. Moreover, its network parameters, computational demands, and model size exceed those of YOLOv7-SPSD.

**Figure 12:**
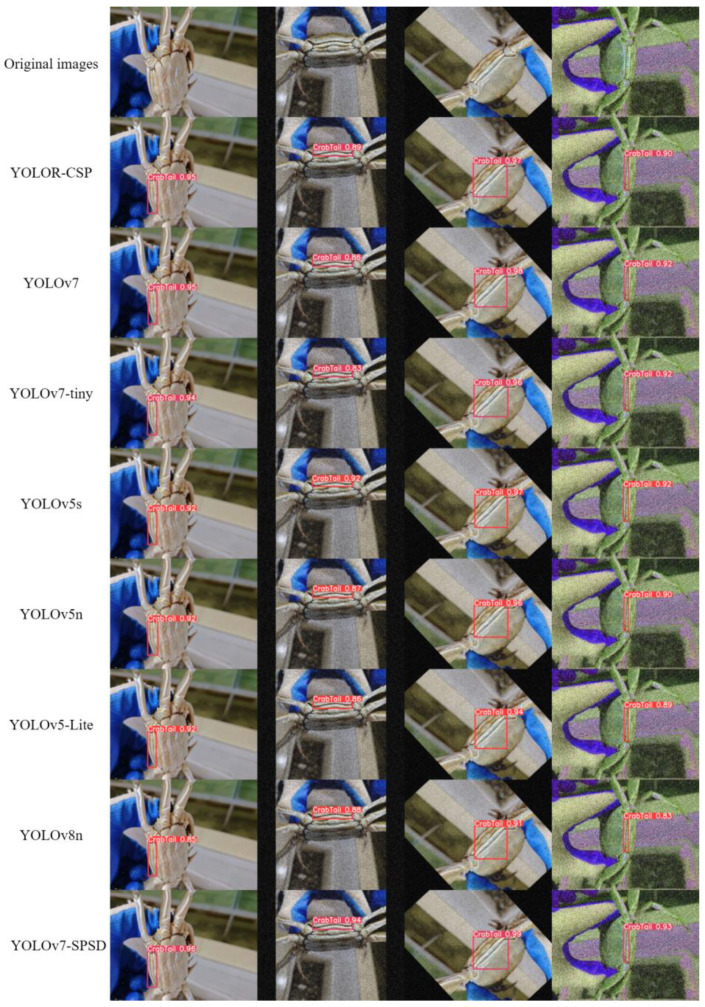
Graph of detection effect of different models.

The enhanced and pruned YOLOv7-SPSD model demonstrates significant efficiency improvements over YOLOv7-tiny, with parameters reduced by 71.6%, GFLOPS by 74.6%, and size by 69.2%. Additionally, it exhibits slight improvements in performance metrics, with precision, mAP0.5, and F1-score increasing by 0.1%, and recall by 0.2%. Clearly, the YOLOv7-SPSD model significantly reduces the computational load while maintaining high performance levels. The detection results depicted in Figure 12 indicate that, although the YOLOv5 series and YOLOv8 are slightly less effective than the YOLOv7 series, the YOLOv7-SPSD achieves comparable, and in some complex environments, superior performance to YOLOv7 and YOLOR-CSP. Additionally, its detection accuracy surpasses that of other smaller models, expanding its potential for broader applications.

## 4. Conclusions

This study focuses on the precise part-based processing of river crabs, specifically the crab tail, which is crucial for removing dorsal armor. We propose the YOLOv7-SPSD lightweight deep learning model to efficiently identify and locate the key processing areas of river crabs. The YOLOv7-SPSD algorithm enhances the YOLOv7-tiny framework by incorporating several advancements, including the lightweight Slimneck module, PConv, and SimAM attention mechanisms. Additionally, we have refined this approach by implementing the DepGraph pruning method. This combination effectively merges modular enhancements with advanced pruning techniques.

Our main findings are as follows: The performance of the YOLOv7-SPSD model, enhanced through modularization and pruning, has seen significant improvements. Specifically, mAP0.5 has increased by 0.1%, R by 0.2%, and F1-score by 0.1%, while FPS has improved by 2.4. Furthermore, GFLOPS have been reduced by 74.6%, size by 8.1 MB, and parameters by 71.6%. Compared with current mainstream target detection algorithms, the YOLOv7-SPSD model outperforms most lightweight networks in terms of FPS, parameters, GFLOPS, and size. Its detection efficacy matches or even surpasses that of larger networks such as YOLOv7 and YOLOR-CSP. Additionally, pruning experiments demonstrate that when compressed to 2.7 times using the DepGraph algorithm, the model shows a 0.1% increase in performance compared to before pruning. Moreover, the model’s parameters, GFLOPS, and size are lower than those achieved by other pruning algorithms such as L1, Lamp, Slim, and Taylor.

In conclusion, the YOLOv7-SPSD lightweight deep learning model presented in this paper is both lightweight and effective for detection tasks. It introduces an innovative concept for research on lightweight models and allows robots to incorporate machine vision for accurate, part-based processing of river crabs. This approach provides a new method for enhancing the intelligence of equipment used in the deep processing of river crabs.

## Figures and Tables

**Table 1:** Ablation experiment results (S: Slimneck; P: PConv; SM: SimAM; D: DepGraph; ✓: Add the module).

Model	S	P	SM	D	P/%	R/%	mAP0.5/%	F1%	Parameters	GFLOPS	Size/mb
Baseline					99.5	99.3	99.5	99.4	6,007,596	13	11.7
Step1	✓				98.5	98.5	99.5	98.5	5,794,572	12.2	11.4
Step2	✓	✓			99.2	98.3	99.4	98.8	4,499,852	9.1	8.9
Step3	✓	✓	✓		99.2	99.5	99.5	99.4	4,499,852	9.1	8.9
Ours	✓	✓	✓	✓	99.5	99.5	99.6	99.5	1,708,306	3.3	3.6

**Table 2:** Results of pruning compression experiment.

Compression Ratio	mAP0.5/%	Parameters	GFLOPS	Size/MB
1.5	99.6	3,022,111	6	6.1
2	99.6	2,268,534	4.4	4.6
2.2	99.6	2,085,540	4.1	4.6
2.5	99.6	1,838,419	3.5	4.6
2.7	99.6	1,708,306	3.3	3.6
2.8	99.5	1,633,143	3.1	3.4
3	99.5	1,573,437	3	3.3

**Table 3:** Comparative experimental results of different pruning methods.

Algorithms	Compression Ratio	mAP0.5/%	Parameters	GFLOPS	Size/MB
L1	2.7	99.5	1,708,306	3.3	3.6
Lamp	2.1	99.5	2,177,747	4.2	4.5
Slim	2	99.4	2,268,205	4.4	4.6
Taylor	2.5	99.5	1,838,419	3.5	4.6
DepGraph	2.7	99.6	1,708,306	3.3	3.6

**Table 4:** Comparison results of different network models.

Models	P/%	R/%	mAP0.5/%	F1%	Parameters	GFLOPS	Size/MB	FPS
YOLOR-CSP	99.8	99.5	99.6	99.6	52,465,484	118.9	100.6	1.7
YOLOv7	99	99.7	99.6	99.3	36,481,772	103.2	70.0	1.8
YOLOv7-tiny	99.5	99.3	99.5	99.4	6,007,596	13	11.7	4.7
YOLOv5s	99.3	99.2	99.4	99.2	7,012,822	15.8	13.7	5.6
YOLOv5n	99.1	98.7	99.3	98.9	1,760,518	4.1	3.7	8.4
YOLOv5-Lite	97.5	96.7	97.3	97.1	1,540,854	3.7	3.3	5.9
YOLOv8n	99.5	99.2	99.4	99.3	3,005,843	8.1	6.0	6.6
Ours	99.6	99.5	99.6	99.5	1,708,306	3.3	3.6	7.1

## Data Availability

The data collected in this research are available when required.
